# HER2 expression, copy number variation and survival outcomes in HER2-low non-metastatic breast cancer: an international multicentre cohort study and TCGA-METABRIC analysis

**DOI:** 10.1186/s12916-022-02284-6

**Published:** 2022-03-17

**Authors:** Ryan Shea Ying Cong Tan, Whee Sze Ong, Kyung-Hun Lee, Abner Herbert Lim, Seri Park, Yeon Hee Park, Ching-Hung Lin, Yen-Shen Lu, Makiko Ono, Takayuki Ueno, Yoichi Naito, Tatsuya Onishi, Geok-Hoon Lim, Su-Ming Tan, Han-Byoel Lee, Han Suk Ryu, Wonshik Han, Veronique Kiak Mien Tan, Fuh-Yong Wong, Seock-Ah Im, Puay Hoon Tan, Jason Yongsheng Chan, Yoon-Sim Yap

**Affiliations:** 1 Division of Medical Oncology, National Cancer Centre Singapore, 11 Hospital Crescent, Singapore, 169610 Singapore; 2 Division of Clinical Trials and Epidemiological Sciences, National Cancer Centre Singapore, Singapore, Singapore; 3 Department of Internal Medicine, Seoul National University Hospital, Cancer Research Institute, Seoul National University College of Medicine, Seoul, Republic of Korea; 4 Cancer Discovery Hub, National Cancer Centre Singapore, Singapore, Singapore; 5 Department of Health Sciences and Technology, SAIHST, Sungkyunkwan University, Seoul, Republic of South Korea; 6 Division of Hematology-Oncology, Department of Medicine, Samsung Medical Center, Sungkyunkwan University School of Medicine, Seoul, Republic of South Korea; 7 Department of Oncology, National Taiwan University Hospital, Taipei, Taiwan; 8 Department of Medical Oncology, Cancer Institute Hospital, Japanese Foundation for Cancer Research, Tokyo, Japan; 9 Breast Surgical Oncology, Breast Oncology Center, Cancer Institute Hospital, Japanese Foundation for Cancer Research, Tokyo, Japan; 10 Department of Medical Oncology, National Cancer Center Hospital East, Kashiwa, Chiba Japan; 11 Department of Breast Surgery, National Cancer Center Hospital East, Kashiwa, Chiba Japan; 12 Breast Department, KK Women’s and Children’s Hospital, Singapore, Singapore; 13 Sing Health Duke-NUS Breast Centre, Singapore, Singapore; 14 Division of Breast Surgery, Department of General Surgery, Changi General Hospital, Singapore, Singapore; 15 Department of Surgery, Seoul National University Hospital, Cancer Research Institute, Seoul National University College of Medicine, Seoul, Republic of Korea; 16 Department of Pathology, Seoul National University Hospital, Cancer Research Institute, Seoul National University College of Medicine, Seoul, Republic of Korea; 17 Division of Surgery and Surgical Oncology, National Cancer Centre Singapore, Singapore, Singapore; 18 Department of Breast Surgery, Singapore General Hospital, Singapore, Singapore; 19 Division of Radiation Oncology, National Cancer Centre Singapore, Singapore, Singapore; 20 Division of Pathology, Singapore General Hospital, Singapore, Singapore; 21 Duke-NUS Medical School, Singapore, Singapore

**Keywords:** HER2-low breast cancer, *ERBB2* neutral, Prognosis, TCGA, METABRIC

## Abstract

**Background:**

HER2-low breast cancer (BC) is currently an area of active interest. This study evaluated the impact of low expression of HER2 on survival outcomes in HER2-negative non-metastatic breast cancer (BC).

**Methods:**

Patients with HER2-negative non-metastatic BC from 6 centres within the Asian Breast Cancer Cooperative Group (ABCCG) (*n* = 28,280) were analysed. HER2-low was defined as immunohistochemistry (IHC) 1+ or 2+ and in situ hybridization non-amplified (ISH−) and HER2-zero as IHC 0. Relapse-free survival (RFS) and overall survival (OS) by hormone receptor status and HER2 IHC 0, 1+ and 2+ ISH− status were the main outcomes. A combined TCGA-BRCA and METABRIC cohort (*n* = 1967) was also analysed to explore the association between HER2 expression, *ERBB2* copy number variation (CNV) status and RFS.

**Results:**

ABCCG cohort median follow-up was 6.6 years; there were 12,260 (43.4%) HER2-low BC and 16,020 (56.6%) HER2-zero BC. The outcomes were better in HER2-low BC than in HER2-zero BC (RFS: centre-adjusted hazard ratio (HR) 0.88, 95% CI 0.82–0.93, *P* < 0.001; OS: centre-adjusted HR 0.82, 95% CI 0.76–0.89, *P* < 0.001). On multivariable analysis, HER2-low status was prognostic (RFS: HR 0.90, 95% CI 0.85–0.96, *P* = 0.002; OS: HR 0.86, 95% CI 0.79–0.93, *P* < 0.001). These differences remained significant in hormone receptor-positive tumours and for OS in hormone receptor-negative tumours. Superior outcomes were observed for HER2 IHC1+ BC versus HER2-zero BC (RFS: HR 0.89, 95% CI 0.83–0.96, *P* = 0.001; OS: HR 0.85, 95% CI 0.78–0.93, *P* = 0.001). No significant differences were seen between HER2 IHC2+ ISH− and HER2-zero BCs. In the TCGA-BRCA and METABRIC cohorts, *ERBB2* CNV status was an independent RFS prognostic factor (neutral versus non-neutral HR 0.71, 95% CI 0.59–0.86, *P* < 0.001); no differences in RFS by *ERBB2* mRNA expression levels were found.

**Conclusions:**

HER2-low BC had a superior prognosis compared to HER2-zero BC in the non-metastatic setting, though absolute differences were modest and driven by HER2 IHC 1+ BC. *ERBB2* CNV merits further investigation in HER2-negative BC.

**Supplementary Information:**

The online version contains supplementary material available at 10.1186/s12916-022-02284-6.

## Background

Human epidermal growth factor receptor 2 (HER2) is a transmembrane receptor tyrosine kinase that is over-expressed in 10–30% of invasive breast cancers (BC) [1, 2]. HER2 over-expression, typically defined as HER2 immunohistochemistry (IHC) score 3+ or amplification on in situ hybridization (ISH), is an important predictive biomarker for HER2-targeted therapies [3–5].

Currently, there is interest in a new classification of BCs with low to moderate levels of HER2 expression on IHC staining—intensity 1+ or 2+ with non-amplification ISH (ISH−), termed as HER2-low BCs [6]. In recent phase 1B clinical trials using novel HER2-directed antibody drug conjugates (ADC), this subset of HER2-low BCs achieved response rates of 28–44% [7, 8]. These findings have prompted several additional studies, including ongoing randomized phase 3 trials testing these HER2-directed ADCs in pretreated patients with advanced HER2-low BC [9, 10].

This brings into question if HER2-low should represent a separate subtype of BC distinct from HER2-zero (IHC score 0) tumours, as this would have wide-ranging implications from HER2 testing algorithms to clinical trial design. Another consideration is whether there are prognostic differences between these two groups in early-stage BC. However, studies currently present conflicting results. While a recent pooled analysis of 2310 patients from 4 neoadjuvant clinical trials showed better disease-free survival (DFS) and overall survival (OS) in HER2-low BC [11], other studies in the non-metastatic setting [12–14] and metastatic setting [15–17] did not observe any significant differences. In contrast, two older studies reported inferior DFS in non-metastatic BCs that were HER2 IHC 2+ ISH− compared with those which were HER2 IHC 1+ or 0 [18, 19]. A recent study had also reported an increased risk of brain metastasis and inferior DFS of hormone receptor-positive HER2-low compared to hormone receptor-positive HER2-zero localized BC [20].

To address this question, our study aimed to compare the relapse-free survival (RFS) and OS of HER2-low tumours with HER2-zero tumours by hormone receptor status and by HER2 IHC 0, 1+ and 2+ ISH− status in a large multicentre cohort of non-metastatic BC patients. The Cancer Genome Atlas Breast Invasive Carcinoma (TCGA-BRCA) and Molecular Taxonomy of Breast Cancer International Consortium (METABRIC) datasets were analysed to investigate the association of HER2 expression according to IHC, mRNA expression and discrete copy number variation (CNV) with RFS [21, 22].

## Methods

### Study cohort and design

Female patients diagnosed with stage I–III BC between 1 January 2000 and 31 December 2015 and who underwent primary breast surgery were identified from prospectively maintained breast cancer registries in six academic institutions within the Asian Breast Cancer Cooperative Group (ABCCG). Patients with positive, indeterminate or missing HER2 status, or who lacked follow-up information after diagnosis or surgery, were excluded. Details of data from each study centre are summarized (Additional file 1: Table S1). The study was approved by the Singapore Health Services’ Institutional Review Board (CIRB Ref: 2019/2419) and the respective ethics committees in the participating institutions.

### Variables and outcome measures

Extracted information included patient demographics, tumour characteristics (including estrogen receptor [ER], progesterone receptor [PR], HER2 IHC and HER2 ISH status based on the prevailing American Society of Clinical Oncology/College of American Pathologists [ASCO-CAP] recommendations) [23–26] and treatment administered [27]. HER2-positive was defined as IHC score of 3+ or ISH amplified, HER2-zero was defined as IHC score of 0 while HER2-low was defined as IHC score of 1+ or 2+ and ISH−. Details of HER2 antibodies and detection systems are listed (Additional file 1: Table S1). Pathology laboratories at all institutions were accredited by CAP or the national pathology accreditation body and adopted ASCO-CAP guidelines of 2007 and 2013 for HER2 testing within 3 months of publication [19, 20]. All BCs were staged pathologically according to the 5th, 6th or 7th edition of TNM classification by the American Joint Committee on Cancer (AJCC), which were generally adopted within 3 months after publication [28–30]. Clinical staging was used for patients that received neoadjuvant therapy. Outcome measures were RFS and OS, each defined according to STEEP version 2 [31].

### Statistical analyses

Categorical characteristics were compared between HER2-zero and HER2-low patients using Fisher’s exact test. Follow-up duration was estimated using the reverse Kaplan-Meier method. RFS and OS were estimated using the Kaplan-Meier method. The association of each survival outcome with each characteristic was assessed via Cox proportional hazard (PH) model and tested using Wald’s test. PH assumption was verified based on Schoenfeld residuals. Study centre was included as a covariate in each Cox model to account for the heterogeneity of survival outcomes across study centres. Heterogeneity was assessed using the index of heterogeneity (*I*^2^), which was generated by pooling the univariable hazard ratio (HR) estimate from each study centre with a random effect restricted maximum likelihood estimation model. Multivariable Cox models included HER2 status, study centre, age at diagnosis, ethnicity, year of diagnosis, histology, overall stage, hormone receptor status, grade, radiotherapy, endocrine therapy and chemotherapy. Hormone receptor status subgroup analyses were conducted by including a HER2 status and hormone receptor status interaction term in the Cox model. All models were fitted using the entire cohort; no imputation for missing values was performed.

Additional sensitivity analyses were performed to (a) account for the heterogeneity between study centres alternatively via multilevel Cox model and (b) assess the impact of the longer follow-up duration among the HER2-zero than HER2-low patients on their survival outcomes. For (a), each study centre was deemed as a cluster of patients and adjusted as such in the Cox model. For (b), HER2-zero patients had longer follow-up duration as there was a higher percentage of these patients diagnosed between 2000 and 2010. Patients diagnosed in 2000–2010 were censored at the maximum follow-up time of patients diagnosed in 2011–2015 (9.8 years).

### Bioinformatic analyses of TCGA-BRCA and METABRIC data

Cases diagnosed with stage I–III HER2-negative BC in the METABRIC and TCGA-BRCA datasets were extracted from cBioPortal for analysis (*n* = 1967) [21, 22, 32, 33]. Additionally, *z*-score transformed *ERBB2* mRNA expression levels, *ERBB2* CNV, intrinsic subtype classifications and RFS data were extracted. HER2 IHC details were available only for the TCGA-BRCA cohort. Discrete *ERBB2* CNV status was based on the Genomic Identification of Significant Targets in Cancer (GISTIC) method (− 2, loss of both copies; − 1, one copy loss; 0, neutral; 1, low-level gain [a few additional copies, often broad]; 2, high-level amplification [more copies, often focal]), and *z*-score transformed mRNA expression implied a relative expression level compared to the average mRNA expression across the patients (https://docs.cbioportal.org/1.-general/faq) [34]. *ERBB2* mRNA expression by HER2 IHC scores and *ERBB2* CNV were compared using the Kruskal-Wallis test, and their correlation was assessed using the Spearman correlation coefficient. The frequency of intrinsic subtypes (PAM50 classification for TCGA-BRCA; PAM50 + claudin-low classification for METABRIC) by *ERBB2* CNV was compared using Fisher’s exact test. Kaplan-Meier curves of RFS were compared using the log-rank test. Covariates included in the multivariable Cox model were *ERBB2* CNV status, grade, stage, hormone receptor status and patient’s age.

Analyses were performed using SAS version 9.4 (SAS Institute Inc., Cary, NC), Stata version 16 (StataCorp, College Station, TX) and MedCalc for Windows version 19.0.4 (MedCalc Software, Ostend, Belgium). All statistical tests were 2-sided with a 5% significance level.

## Results

### Analysis of ABCCG cohort

A total of 38,853 patients were identified in the combined dataset, of which 7503 (19.3%) were HER2-positive, 3022 had missing or indeterminate HER2 status and 48 lacked follow-up information (Additional file 1: Fig. S1). The remaining 28,280 patients were analysed: 12,260 (43.4%) had HER2-low tumours, and 16,020 (56.6%) were HER2-zero. Clinicopathological characteristics by HER2 and hormone receptor status are shown in Table 1.

**Table 1:** Clinicopathological features by HER2 and hormone receptor status

	Total (%)			Hormone receptor-positive (%)			Hormone receptor-negative (%)		
	HER2-zero (***n*** = 16,020)	HER2low (***n*** = 12,260)	***P***	HER2-zero (***n*** = 12,712)	HER2-low (***n*** = 10,791)	***P***	HER2-zero (***n*** = 3272)	HER2-low (***n*** = 1362)	***P***
Age at diagnosis, years									
Below 35	787 (4.9)	541 (4.4)	0.071	528 (4.2)	455 (4.2)	0.014	256 (7.8)	77 (5.7)	< 0.001
35–49	7448 (46.5)	5798 (47.3)		6131 (48.2)	5244 (48.6)		1291 (39.5)	466 (34.2)	
50–64	5639 (35.2)	4357 (35.5)		4319 (34.0)	3768 (34.9)		1315 (40.2)	581 (42.7)	
65 and over	2146 (13.4)	1564 (12.8)		1734 (13.6)	1324 (12.3)		410 (12.5)	238 (17.5)	
Ethnicity									
Chinese	5203 (32.5)	4674 (38.1)	< 0.001	4344 (34.2)	4183 (38.8)	< 0.001	853 (26.1)	483 (35.5)	< 0.001
Malay	374 (2.3)	220 (1.8)		301 (2.4)	194 (1.8)		73 (2.2)	26 (1.9)	
Indian	253 (1.6)	149 (1.2)		191 (1.5)	123 (1.1)		62 (1.9)	26 (1.9)	
Korean	6873 (42.9)	5363 (43.7)		5245 (41.3)	4739 (43.9)		1622 (49.6)	613 (45.0)	
Japanese	3182 (19.9)	1782 (14.5)		2516 (19.8)	1488 (13.8)		642 (19.6)	206 (15.1)	
Others	135 (0.8)	72 (0.6)		115 (0.9)	64 (0.6)		20 (0.6)	8 (0.6)	
Year of diagnosis									
2000–2005	2171 (13.6)	906 (7.4)	< 0.001	1697 (13.4)	714 (6.6)	< 0.001	466 (14.2)	172 (12.6)	0.002
2006–2010	6501 (40.6)	3835 (31.3)		5069 (39.9)	3286 (30.5)		1421 (43.4)	538 (39.5)	
2011–2015	7348 (45.9)	7519 (61.3)		5946 (46.8)	6791 (62.9)		1385 (42.3)	652 (47.9)	
Histology									
IDC^a^	14,090 (88.0)	11,043 (90.1)	< 0.001	11,019 (86.7)	9665 (89.6)	< 0.001	3040 (92.9)	1277 (93.8)	0.323
ILC^a^	895 (5.6)	688 (5.6)		821 (6.5)	654 (6.1)		72 (2.2)	32 (2.4)	
Others	1035 (6.5)	529 (4.3)		872 (6.9)	472 (4.4)		160 (4.9)	53 (3.9)	
T-stage									
T1	8828 (55.1)	6815 (55.6)	0.003	7413 (58.3)	6112 (56.6)	0.002	1392 (42.5)	635 (46.6)	0.100
T2	6011 (37.5)	4629 (37.8)		4472 (35.2)	4008 (37.1)		1528 (46.7)	594 (43.6)	
T3	719 (4.5)	557 (4.5)		528 (4.2)	478 (4.4)		189 (5.8)	75 (5.5)	
T4	309 (1.9)	167 (1.4)		209 (1.6)	131 (1.2)		100 (3.1)	30 (2.2)	
Others^b^	103 (0.6)	58 (0.5)		46 (0.4)	30 (0.3)		57 (1.7)	27 (2.0)	
Unknown	50 (0.3)	34 (0.3)		44 (0.4)	32 (0.3)		6 (0.2)	1 (0.1)	
N-stage									
N0	10,412 (65.0)	7679 (62.6)	0.001	8199 (64.5)	6754 (62.6)	0.042	2191 (67.0)	855 (62.8)	0.005
N1	3865 (24.1)	3101 (25.3)		3118 (24.5)	2760 (25.6)		735 (22.5)	319 (23.4)	
N2	1081 (6.8)	920 (7.5)		887 (7.0)	810 (7.5)		194 (5.9)	102 (7.5)	
N3	647 (4.0)	543 (4.4)		494 (3.9)	453 (4.2)		151 (4.6)	83 (6.1)	
NX	15 (0.1)	17 (0.1)		14 (0.1)	14 (0.1)		1 (0.0)	3 (0.2)	
Overall stage									
Stage 1	6782 (42.3)	5217 (42.6)	0.159	5732 (45.1)	4712 (43.7)	0.050	1034 (31.6)	452 (33.2)	0.033
Stage 2	6881 (43.0)	5154 (42.0)		5206 (41.0)	4485 (41.6)		1657 (50.6)	636 (46.7)	
Stage 3	2357 (14.7)	1889 (15.4)		1774 (14.0)	1594 (14.8)		581 (17.8)	274 (20.1)	
ER receptor status									
Positive	12,338 (77.0)	10,578 (86.3)	< .0001	12,338 (97.1)	10,578 (98.0)	< 0.001	0 (–)	0 (–)	-
Negative	3643 (22.7)	1574 (12.8)		371 (2.9)	210 (2.0)		3272 (100)	1362 (100)	
Unknown^c^	39 (0.2)	108 (0.9)		3 (0.0)	3 (0.0)		0 (–)	0 (–)	
PR receptor status									
Positive	10,832 (67.6)	9296 (75.8)	< 0.001	10,832 (85.2)	9296 (86.2)	0.050	0 (–)	0 (–)	–
Negative	5136 (32.1)	2853 (23.3)		1864 (14.7)	1488 (13.8)		3272 (100)	1362 (100)	
Unknown^b^	52 (0.3)	111 (0.9)		16 (0.1)	7 (0.1)		0 (–)	0 (–)	
Tumour grade									
Grade 1	3586 (22.4)	2745 (22.4)	< 0.001	3471 (27.3)	2657 (24.6)	< 0.001	104 (3.2)	47 (3.5)	0.028
Grade 2	6544 (40.9)	5601 (45.7)		5882 (46.3)	5277 (48.9)		648 (19.8)	291 (21.4)	
Grade 3	4879 (30.5)	3219 (26.3)		2552 (20.1)	2296 (21.3)		2321 (70.9)	914 (67.1)	
Unknown	1011 (6.3)	695 (5.7)		807 (6.4)	561 (5.2)		199 (6.1)	110 (8.1)	
Received radiotherapy^d^									
Applicable—no	2487 (15.5)	1957 (16.0)	< 0.001	2045 (16.1)	1742 (16.1)	< 0.001	432 (13.2)	189 (13.9)	0.267
Applicable—yes	6302 (39.3)	4114 (33.6)		5015 (39.5)	3583 (33.2)		1272 (38.9)	495 (36.3)	
Not applicable	7231 (45.1)	6189 (50.5)		5652 (44.5)	5466 (50.7)		1568 (47.9)	678 (49.8)	
Received endocrine therapy^c,d^									
Applicable—no	1214 (7.6)	978 (8.0)	< 0.001	1214 (9.6)	978 (9.1)	0.208	0 (–)	0 (–)	–
Applicable—yes	11,498 (71.8)	9813 (80.0)		11,498 (90.5)	9813 (90.9)		0 (–)	0 (–)	
Not applicable	3272 (20.4)	1362 (11.1)		0 (–)	0 (–)		3272 (100)	1362 (100)	
Unknown	36 (0.2)	107 (0.9)		0 (–)	0 (–)		0 (–)	0 (–)	
Received chemotherapy^c,d^									
Applicable—no	2065 (12.9)	1624 (13.3)	0.677	1760 (13.9)	1446 (13.4)	0.003	296 (9.1)	154 (11.3)	0.048
Applicable—yes	7507 (46.9)	5744 (46.9)		5455 (42.9)	4879 (45.2)		2041 (62.4)	836 (61.4)	
Not applicable	5695 (35.6)	4297 (35.1)		4925 (38.7)	3959 (36.7)		756 (23.1)	287 (21.1)	
Unknown	753 (4.7)	595 (4.9)		572 (4.5)	507 (4.7)		179 (5.5)	85 (6.2)	

^a^ *IDC* invasive ductal carcinoma, *ILC* invasive lobular carcinoma

^b^ *TX* Tis and T0

^c^ Included 4 patients with equivocal test result; combined with an unknown category for analysis due to small sample size

^d^ Patients were deemed applicable for radiotherapy if the N-stage was N1–3, T-stage was T3–4 or they had undergone breast-conserving surgery. They were deemed applicable for endocrine therapy if the tumour was ER- or PR-positive. They were deemed applicable for chemotherapy if the N-stage was N1–3, T-stage was T2–4 or tumour size ≥ 10 mm and had any of following risk factors: Grade 3 tumour, ER-negative or HER2-positive. These criteria were adapted from our previous study [27]

While statistically significant differences between HER2-low and HER2-zero tumours were detected for several of the variables, the more prominent differences included the higher percentage of HER2-low tumours in the hormone receptor-positive subgroup than the hormone receptor-negative subgroup (45.9% vs 29.4%, *P* < 0.001) and among the hormone receptor-positive BCs tumours diagnosed more recently during 2011–2015 (62.9% vs 46.8%).

**Fig. 1:**
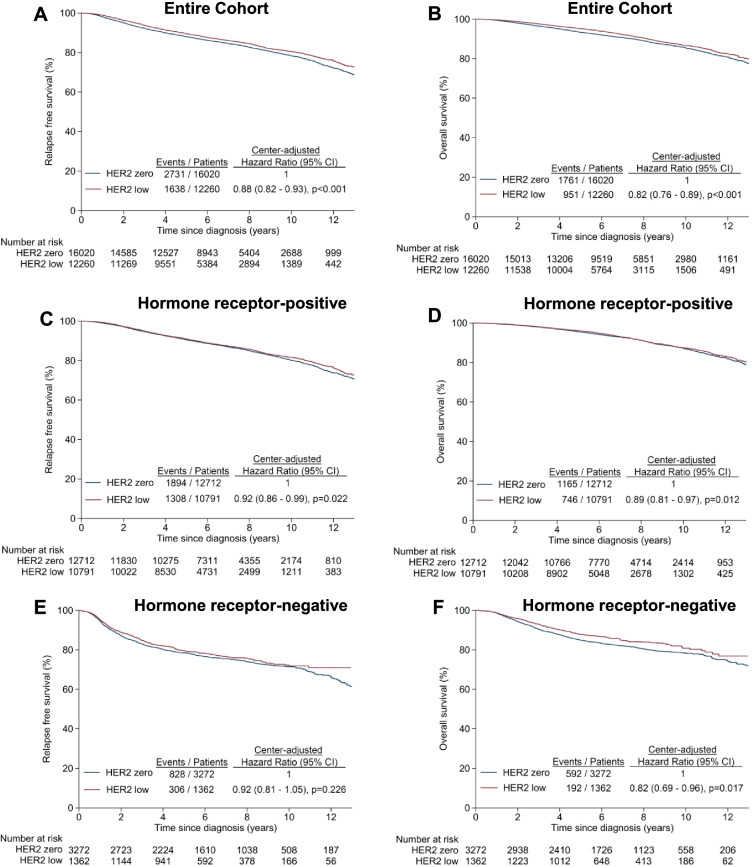
Kaplan-Meier curves of relapse-free survival and overall survival by HER2-low and HER2-zero status. **A**, **B** Entire cohort. **C**, **D** Hormone receptor-positive BC patients. **E**, **F** Hormone receptor-negative BC patients

The median follow-up was 6.6 years (interquartile range [IQR] 4.9–9.1 years). Compared with HER2-zero tumours, HER2-low BC had significantly better RFS (centre-adjusted HR 0.88, 95% CI 0.82–0.93, *P* < 0.001) and OS (centre-adjusted HR 0.82, 95% CI 0.76–0.89, *P* < 0.001) (Fig. 1A, B). This was also observed in the hormone receptor-positive subgroup (RFS: centre-adjusted HR 0.92, 95% CI 0.86–0.99, *P* = 0.022; OS: centre-adjusted HR 0.89, 95% CI 0.81–0.97, *P* = 0.012) (Fig. 1C, D). In the hormone receptor-negative subgroup, HER2-low BC had a non-significant trend towards better RFS (centre-adjusted HR 0.92, 95% CI 0.81–1.05, *P* = 0.226) and significantly longer OS (centre-adjusted HR 0.82, 95% CI 0.69–0.96, *P* = 0.017) than HER2-zero BC (Fig. 1E, F). The association between each survival outcome and HER2 status was not dependent on hormone receptor status, as no significant interaction effects between HER2 and hormone receptor status were observed (Table 2). The results of multivariable analyses were consistent with all corresponding centre-adjusted analyses.

**Table 2:** Cox regression analyses of relapse-free survival and overall survival by HER2 and hormone receptor status

	Centre-adjusted			Multivariable-adjusted^**a**^		
	Hazard ratio (95% CI)	***P***	***P(int)***	Hazard ratio (95% CI)	***P***	***P(int)***
**Relapse-free survival**						
Overall: HER2-low vs HER2-zero	0.88 (0.82–0.93)	< 0.001	–	0.90 (0.85–0.96)	0.002	–
Hormone receptor-positive: HER2-low vs HER2-zero	0.92 (0.86–0.99)	0.022	0.973	0.90 (0.84–0.97)	0.004	0.715
Hormone receptor-negative: HER2-low vs HER2-zero	0.92 (0.81–1.05)	0.226		0.92 (0.81–1.05)	0.244	
Overall: HER2 IHC1+ vs HER2-zero	0.85 (0.79–0.91)	< 0.001	**–**	0.89 (0.83–0.96)	0.001	**–**
Overall: HER2 IHC2+ ISH− vs HER2-zero	0.96 (0.87–1.06)	0.421		0.94 (0.85–1.05)	0.280	
Hormone receptor-positive: HER2 IHC1+ vs HER2-zero	0.89 (0.82–0.96)	0.004	0.962	0.89 (0.82–0.96)	0.003	0.934
Hormone receptor-positive: HER2 IHC2+ ISH− vs HER2-zero	1.01 (0.90–1.13)	0.898		0.94 (0.83–1.05)	0.280	
Hormone receptor-negative: HER2 IHC1+ vs HER2-zero	0.88 (0.76–1.03)	0.107		0.91 (0.78–1.05)	0.206	
Hormone receptor-negative: HER2 IHC2+ ISH− vs HER2-zero	1.04 (0.83–1.30)	0.735		0.97 (0.78–1.22)	0.807	
**Overall survival**						
Overall: HER2-low vs HER2-zero	0.82 (0.76–0.89)	< 0.001	**–**	0.86 (0.79–0.93)	< 0.001	**–**
Hormone receptor-positive: HER2-low vs HER2-zero	0.89 (0.81–0.97)	0.012	0.401	0.87 (0.79–0.96)	0.004	0.538
Hormone receptor-negative: HER2-low vs HER2-zero	0.82 (0.69–0.96)	0.017		0.82 (0.70–0.97)	0.018	
Overall: HER2 IHC1+ vs HER2-zero	0.79 (0.73–0.87)	< 0.001	**–**	0.85 (0.78–0.93)	0.001	**–**
Overall: HER2 IHC2+ ISH− vs HER2-zero	0.91 (0.79–1.04)	0.148		0.88 (0.76–1.01)	0.062	
Hormone receptor-positive: HER2 IHC1+ vs HER2-zero	0.86 (0.78–0.96)	0.005	0.627	0.86 (0.78–0.95)	0.004	0.811
Hormone receptor-positive: HER2 IHC2+ ISH− vs HER2-zero	0.96 (0.82–1.12)	0.632		0.89 (0.76–1.05)	0.171	
Hormone receptor-negative: HER2 IHC1+ vs HER2-zero	0.78 (0.65–0.94)	0.009		0.82 (0.68–0.99)	0.035	
Hormone receptor-negative: HER2 IHC2+ ISH− vs HER2-zero	0.94 (0.71–1.24)	0.654		0.83 (0.62–1.09)	0.178	

*int* interaction between HER2 and hormone-receptor status

^a^Covariates adjusted were study centre, age at diagnosis, ethnicity, year of diagnosis, histology, overall stage, hormone-receptor status, grade, received radiotherapy, endocrine therapy and chemotherapy

**Fig. 2:**
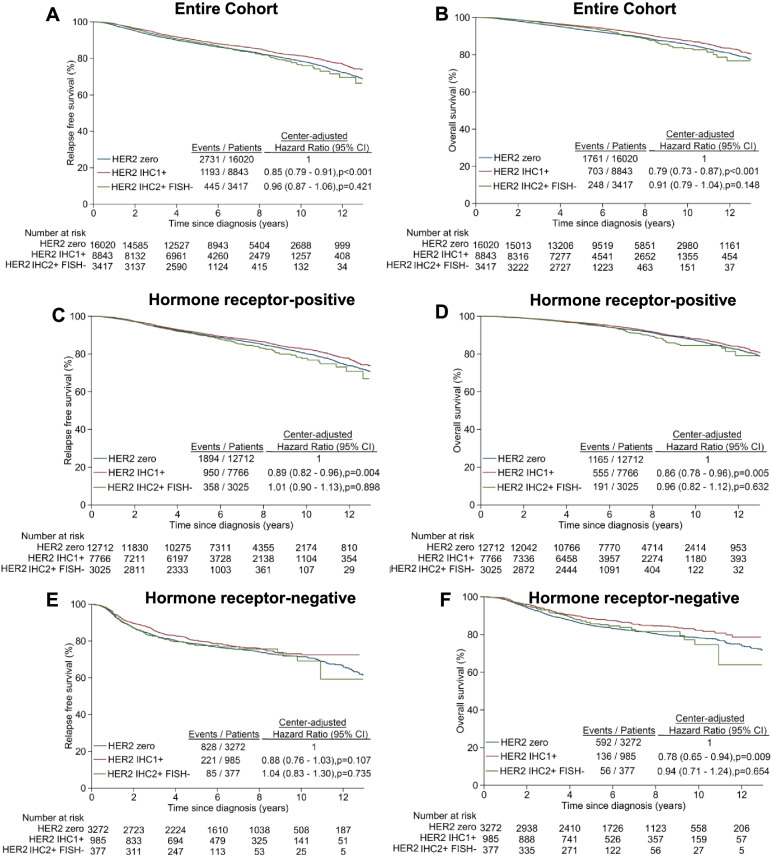
Kaplan-Meier curves of relapse-free survival and overall survival by HER2 IHC score. **A**, **B** Entire cohort. **C**, **D** Hormone receptor-positive BC patients. **E**, **F** Hormone receptor-negative BC patients

There were significant differences in RFS and OS between patients with HER2 IHC 1+ and HER2-zero tumours, but not between those with HER2 IHC 2+ ISH− and HER2-zero tumours (Fig. 2). HER2 IHC 1+ subgroup had longer RFS (centre-adjusted HR 0.85, 95% CI 0.79–0.91, *P* < 0.001) and OS (centre-adjusted HR 0.79, 95% CI 0.73–0.87, *P* < 0.001) than HER2-zero subgroup (Fig. 2A, B). Similar to HER2-low versus HER2-zero comparisons, significant survival differences between HER2 IHC 1+ and HER2-zero were again observed in the hormone receptor-positive subgroup (Fig. 2C, D) and for OS in the hormone receptor-negative subgroup (Fig. 2E, F).

There was moderate heterogeneity between the study centres (RFS *I*^2^ = 63% and OS *I*^2^ = 74%) (Additional file 1: Fig. S2). When heterogeneity between the study centres was accounted alternatively via the multilevel Cox model, no appreciable differences in the resulting HR estimates were observed when compared to those from the original analyses (results not shown). The results of the sensitivity analysis in which patients diagnosed in 2000–2010 were censored at the maximum follow-up time of patients diagnosed in 2011–2015 were broadly similar to the original analyses (Additional file 1: Table S2). HER2-low patients continued to have better RFS and OS than HER2-zero patients overall and within each hormone receptor subgroup (i.e., all centre- and multivariable-adjusted HR were < 1.0), although the OS difference by HER2 status amongst the hormone receptor-positive subgroup was no longer statistically significant.

### Analysis of TCGA-BRCA and METABRIC datasets

**Fig. 3:**
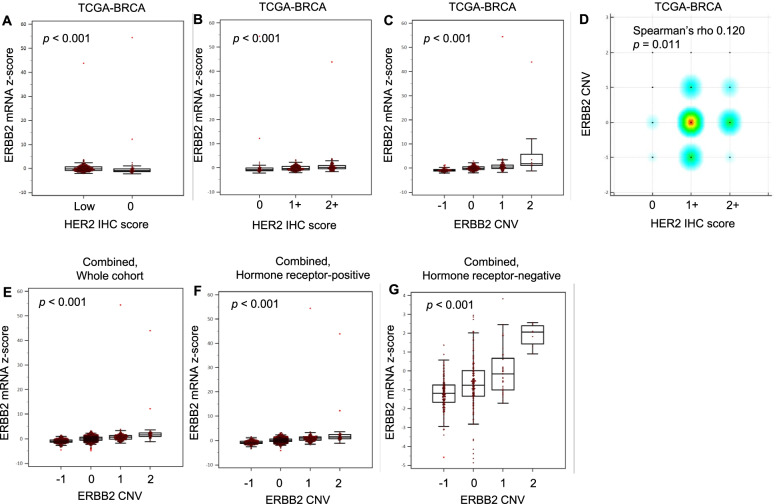
Association of *ERBB2* mRNA expression with HER2 IHC (TCGA-BRCA) and *ERBB2* CNV (TCGA-BRCA; combined dataset). **A**–**D** TCGA-BRCA dataset: *ERBB2* mRNA expression by **A** HER2-low and HER2-zero, **B** HER2 IHC score, **C** *ERBB2* CNV, and **D** *ERBB2* CNV against HER2 IHC. **E**–**G** Combined TCGA-BRCA and METABRIC dataset: *ERBB2* mRNA expression by *ERBB2* CNV among the **E** whole cohort, **F** hormone receptor-positive subgroup, and **G** hormone receptor-negative subgroup

A total of 440 cases were extracted from the TGCA-BRCA database: 58.0% were IHC 1+, 28.9% IHC 2+ ISH−, while the remaining 13.2% were HER2-zero. There were significant differences in *ERBB2* mRNA expression levels by HER2 IHC scores (*P* < 0.001) and *ERBB2* CNV scores (*P* < 0.001) (Fig. 3). Boxplots showed that the higher the *ERBB2* mRNA expression levels, the higher the HER2 IHC scores and *ERBB2* CNV scores. A weak positive correlation was observed between HER2 IHC and CNV scores (Spearman’s rho 0.120, *P* = 0.011); 130 of the 255 (51.0%) IHC 1+ BCs and 69 of the 127 (54.3%) IHC 2+ ISH− BCs were CNV neutral, compared with 25 of the 58 (43.1%) HER2-zero BCs (*P* = 0.3661) (Fig. 3). Findings were similar in hormone receptor-positive (*n* = 356) and hormone receptor-negative (*n* = 84) subgroups, except that no significant correlation was found between HER2 IHC and CNV scores in the latter subgroup (Additional file 1: Fig. S3). Analysis of the combined TCGA-BRCA and METABRIC dataset (*n* = 1967) showed that *ERBB2* CNV scores increased with mRNA expression levels, including both hormone receptor-positive and hormone receptor-negative subgroups (all *P* < 0.001) (Fig. 3). These findings suggest that *ERBB2* gene expression levels may be influenced by copy number alteration in these subsets of breast cancer.

**Fig. 4:**
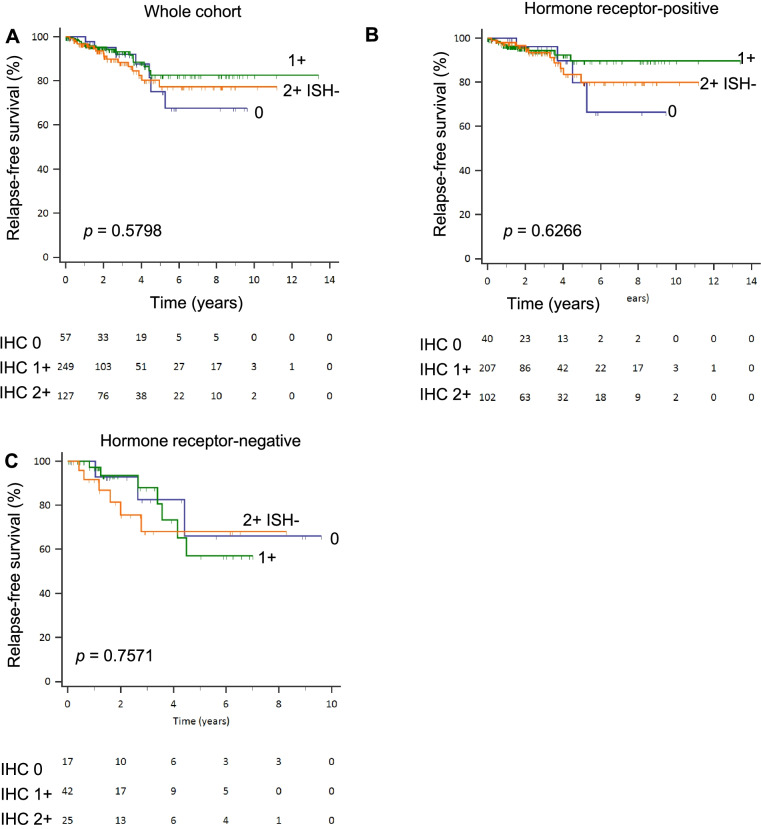
Relapse-free survival by HER2 IHC scores in TCGA-BRCA dataset. **A** Whole cohort. **B** Hormone receptor-positive subgroup. **C** Hormone receptor-negative subgroup

**Fig. 5:**
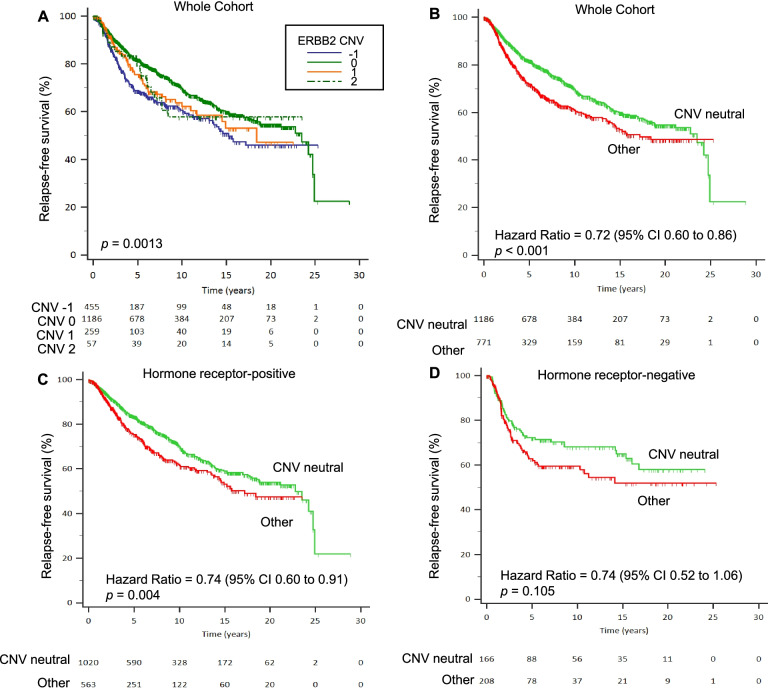
Relapse-free survival in combined TCGA-BRCA and METABRIC dataset. **A** *ERBB2* CNV based on GISTIC definition in the entire cohort. **B** *ERBB2* CNV neutral vs non-neutral (other) status in the entire cohort. **C** *ERBB2* CNV neutral vs non-neutral status in hormone receptor-positive subgroup. **D** *ERBB2* CNV neutral vs non-neutral status in hormone receptor-negative subgroup

**Fig. 6:**
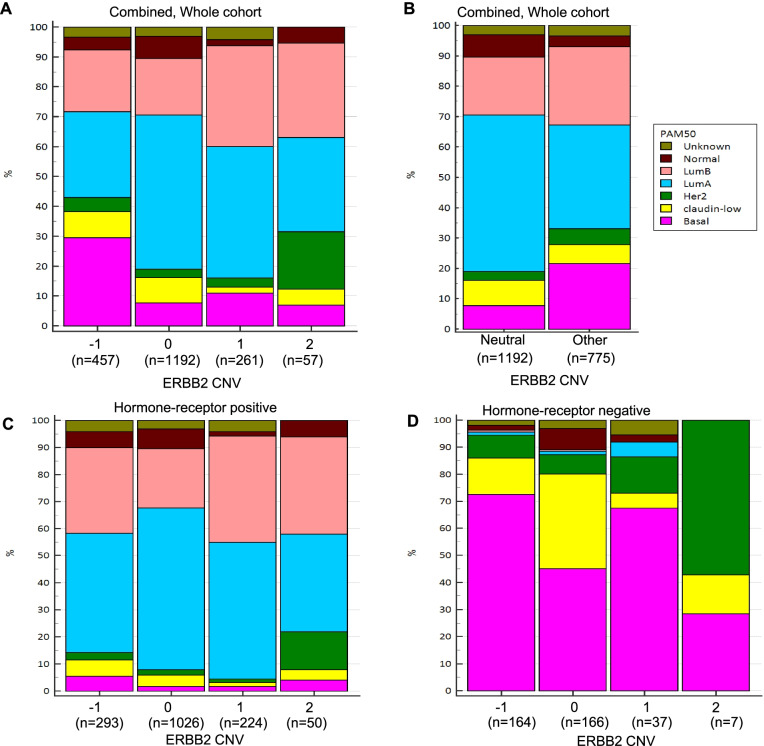
Distribution of intrinsic subtypes with PAM50 and claudin-low classification in combined TCGA-BRCA and METABRIC dataset. **A** *ERBB2* CNV in the entire cohort. **B** *ERBB2* CNV neutral vs non-neutral (other) in the entire cohort. **C** *ERBB2* CNV in hormone receptor-positive subgroup. **D** *ERBB2* CNV in hormone receptor-negative subgroup

Similar to the findings in the ABCCG cohort, HER2 IHC 1+ demonstrated a trend towards better RFS compared to IHC 2+ and HER2-zero in the TCGA-BRCA overall cohort and hormone receptor-positive subgroup (Fig. 4). Given that HER2 IHC scores were able to stratify survival outcomes in the ABCCG cohort, we performed an exploratory analysis of *ERBB2* CNV scores and survival using the combined TCGA-BRCA and METABRIC datasets. Specifically, we hypothesized that *ERBB2* CNV neutrality may confer a better prognosis. Indeed, there were significant differences in RFS by *ERBB2* CNV scores, with *ERBB2* CNV neutral BCs demonstrating superior RFS compared with *ERBB2* CNV non-neutral cases (HR 0.72, 95% CI 0.60–0.86; *P* = 0.001). This was similarly observed in both hormone receptor-positive and hormone receptor-negative subgroups, although the difference was not statistically significant in the latter (Fig. 5). In multivariable analysis, *ERBB2* CNV neutrality remained an independent prognostic factor for RFS (HR 0.71, 95% CI 0.59–0.86, *P* < 0.001). Using available intrinsic subtype classification data, we found that “luminal A” subtype was the most common in *ERBB2* CNV neutral with a frequency of 51.5%, compared to 34.1% in *ERBB2* CNV non-neutral BCs (*P* < 0.001). A similar pattern was observed in the hormone receptor-positive subgroup (“luminal A” subtype: 59.6% vs 45.6%, *P* < 0.001). In the hormone receptor-negative subgroup, “claudin-low” subtype was enriched in *ERBB2* CNV neutral BCs as compared to non-neutral BCs (34.9% vs 12.0%, *P* < 0.001), whereas “basal” subtype was less prominent (45.2% vs 70.2%, *P* < 0.001) (Fig. 6). No significant differences in RFS by *ERBB2* mRNA expression levels were found (Additional file 1: Fig. S4).

## Discussion

This study on HER2-low BCs evaluated the largest real-world cohort to date. We found that HER2-low BCs were more frequent in hormone receptor-positive tumours, similar to other studies [11, 15, 35]. RFS and OS were superior for HER2-low compared to HER2-zero non-metastatic BC. Statistically significant survival differences were seen in both hormone receptor subgroups, but absolute differences were modest and not clinically significant enough to justify de-escalation of treatment. Notably, the higher RFS and OS were driven by the HER2 IHC 1+ and not IHC 2+ ISH− subgroup in the ABCCG cohort.

These findings are partly consistent with the recent pooled analysis by Denkert et al (*n* = 2310) which showed better DFS and OS in HER2-low early-stage BC compared to HER2-zero BC [11]. However, survival differences were observed only in the hormone receptor-negative subgroup in that study. This may be related to the different endpoint used and shorter follow-up duration (median 46∙6 months [IQR 35∙0–52∙3]). We chose to analyse RFS instead of DFS to focus on BC relapse events; new breast or non-breast primary cancers were not included as events. The survival differences in the hormone receptor-positive subgroup emerged with longer follow-up after 6 years in our study. Survival curves diverged earlier for the hormone receptor-negative subset, consistent with the tendency of triple-negative tumours to relapse early. The OS difference in the hormone receptor-negative subgroup with a non-significant trend in RFS may be confounded by deaths unrelated to BC, or differences in the type of relapse (e.g. distant versus locoregional), which may affect subsequent survival status (Fig. 1). Horisawa et al. recently reported DFS and OS trends in HER2-low versus HER2-zero early BC (*n* =4918) similar to our cohort, except that statistical significance was not reached [14].

Schettini et al. demonstrated that within hormone receptor-positive BCs, expression of *ERBB2* and luminal-related genes was higher in HER2-low BCs compared to HER2-zero; there was no differential expression of genes in hormone receptor-negative BCs according to HER2 expression [15]. Interestingly, in early ER-positive BCs with high genomic risk (OncotypeDx risk score > 25), Mutai et al. observed more favourable outcomes of HER2-low compared to HER2-zero tumours [36]. It is worth noting that in advanced BC, a modestly superior OS (HR 0.95, 95% CI 0.91 to 0.99) was also observed by Frenel et al (*n* = 15,054) for HER2-low BC in a preliminary report [35], particularly for the hormone receptor-negative subgroup, although smaller studies have not detected significant differences [15–17].

An important novel finding was that within the HER2-low subgroup, HER2 IHC 1+ tumours appeared prognostically distinct from HER2 2+ ISH− tumours (Fig. 2). While HER2 IHC 1+ BC had significantly better RFS and OS compared to HER2-zero BC, HER2 2+ ISH− BC did not. This partly supports previous findings by Eggeman et al. (*n* = 5907) and Rossi et al. (*n* = 1150) [18, 19], where patients with HER2 IHC 2+ ISH− early BC had worse DFS than those with HER2 IHC 0 or 1+. In summary, differences in the findings among the various studies published may be influenced by the endpoint(s) analysed, the follow-up duration and the sample size, with statistically significant differences achieved mainly in the larger studies, as well as comparisons between HER2 IHC 1+ and IHC 2+ ISH− BCs. HER2 IHC scoring is also subject to inter-observer variability with different methodologies at different sites.

To look for possible biological explanations, we conducted an in silico analysis of a combined TCGA-BRCA and METABRIC dataset and found that “*ERBB2*-neutral” CNV status independently conferred a better prognosis. While more studies are required, this finding has important implications for future diagnostic algorithms given the inherent challenge for pathologists to distinguish HER2-zero and HER2-low tumours by IHC in clinical practice. It also warrants further study for a general understanding of HER2-low disease biology given that HER2 status can drift with treatment [37, 38].

Limitations of our study include its retrospective nature and lack of central pathology review. However, while discordance from inter-observer reproducibility issues, different techniques and antibodies has been described [8, 15, 39, 40], all participating centres adopted ASCO-CAP guidelines for HER2 interpretation, providing a measure of standardization [25, 26, 41]. There were proportionately more HER2-zero patients diagnosed during 2000–2010, as fewer HER2 IHC 2+ tumours were sent for ISH testing in earlier cohorts and had been excluded from the analysis. However, sensitivity analysis showed that this did not distort the main findings. Heterogeneity was another limitation and underscores challenges faced in the real world, although additional sensitivity analysis indicated that our findings after accounting for heterogeneity were robust. While ASCO-CAP guidelines for HER2 testing have been updated over the past two decades, definitions of HER2 IHC 0 and 1+ remain unchanged [25, 26, 41]. Although the exploratory analysis of the combined TCGA-BRCA and METABRIC dataset showing superior RFS in “*ERBB2* neutral” tumours provides additional insight on the differences in outcome among HER2-negative BCs, the number of BCs with both IHC and CNV data in TCGA-BRCA series was limited, especially for HER2-zero and IHC 2+ ISH− tumours. Integration of standardized HER2 IHC scoring with CNV and intrinsic subtype status could be considered for future studies.

## Conclusions

In conclusion, our study has revealed that the clinical entity of HER2-low breast cancer is more complex than what we understand from the existing literature. There is an unmet need to develop better methods to distinguish HER2-low BC for more accurate pathological assessment and treatment selection. We found that HER2-low BC was associated with a better prognosis than HER2-zero BC in the non-metastatic setting, although the absolute differences were relatively modest. While this low expression of HER2 may not be the driver for a distinct subtype such as HER2-overexpressing BCs, differences in outcomes observed may be related to the varying distribution of intrinsic subtypes, or it may serve as a surrogate for genomic stability and other factors. This difference also appeared to be driven by the HER2 IHC 1+ subset, which carried a more favourable prognosis than HER2 IHC 2+ ISH− and HER2-zero BC. “*ERBB2* neutral” status may be a better prognostic factor. The biological significance of HER2 expression by copy number within HER2-negative BCs merits further investigation.

## Supplementary Information

**Additional file 1: Table S1.** Database information and details of HER2 testing by study centre. **Table S2.** Sensitivity analysis of relapse-free survival and overall survival by HER2 and hormone receptor status. **Figure S1.** Flowchart of patients included in the ABCCG cohort analysis. **Figure S2.** Estimated heterogeneity between study centres in (A) relapse-free survival, and (B) overall survival (based on pooling univariable HR estimate from each centre with a random effect restricted maximum likelihood estimation model. HR, hazard ratio; I^2^, index of heterogeneity). **Figure S3.** Association of *ERBB2* mRNA expression with HER2 IHC and *ERBB2* CNV by hormone receptor status (TCGA-BRCA): (A-D) Hormone receptor-positive subgroup of TCGA-BRCA dataset: *ERBB2* mRNA expression by (A) HER2-low and HER2-zero, (B) HER2 IHC score, (C) *ERBB2* CNV; (D) *ERBB2* CNV against HER2 IHC. (E-H) Hormone receptor-negative subgroup of TCGA-BRCA dataset: *ERBB2* mRNA expression by (E) HER2-low and HER2-zero, (F) HER2 IHC score, (G) *ERBB2* CNV; (H) *ERBB2* CNV against HER2 IHC. **Figure S4.** Relapse-free survival by *ERBB2* mRNA expression levels in combined TCGA-BRCA and METABRIC dataset (Quartile 1: lowest; Quartile 4: highest)

## Data Availability

The study team is agreeable to share aggregate participant data that underlie the results reported in this article (after de-identification), upon reasonable request. To gain access, data requestors should submit a request to the corresponding author.

## Abbreviations

ABCCG: Asian Breast Cancer Cooperative Group
AJCC: American Joint Committee on Cancer
ASCO-CAP: American Society of Clinical Oncology/College of American Pathologists
BC: Breast cancer
CNV: Copy number variation
DFS: Disease-free survival
ER: Estrogen receptor
GISTIC: Genomic Identification of Significant Targets in Cancer
HER2: Human epidermal growth factor receptor 2
HER2-zero: Immunohistochemistry score zero
HR: Hazard ratio
*I*^2^: Index of heterogeneity
IHC: Immunohistochemistry (IHC)
ISH: In-situ hybridization
ISH: In-situ hybridization non-amplified
METABRIC: Molecular Taxonomy of Breast Cancer International Consortium
OS: Overall survival
PH: Proportional hazard
PR: Progesterone receptor
RFS: Relapse-free survival
TCGA-BRCA: The Cancer Genome Atlas Breast Invasive Carcinoma
