# Supplementary material for: HER2 expression, copy number variation and survival outcomes in HER2-low non-metastatic breast cancer: an international multicentre cohort study and TCGA-METABRIC analysis
Source: BMC Med. 2022 Mar 17;20:105. doi: 10.1186/s12916-022-02284-6 (PMC8928638; doi:10.1186/s12916-022-02284-6)

**Online Supplementary Material**

**Table S1.** Database information and details of HER2 testing by study center

|  | **Combined** | **JBCR** | **NTUH** | **SMC** | **SNUH** | **JFCR** | **NCCHE** |
| --- | --- | --- | --- | --- | --- | --- | --- |
| **Data available** | 2000-2015 | 2000-2015 | 2005-2015 | 2005-2014 | 2004-2015 | 2004-2015 | 2002-2015 |
| **Data cut-off** | - | Dec 2019 | July 2021 | Nov 2018 | Dec 2019 | Nov 2020 | Jun 2020 |
|  |  |  |  |  |  |  |  |
| **No. of patients (%)** | 28280 (100) | 6716 (100) | 4364 (100) | 4532 (100) | 7704 (100) | 2779 (100) | 2185 (100) |
| HER2-zero | 16020 (57) | 4281 (64) | 1684 (39) | 2695 (59) | 4178 (54) | 1622 (58) | 1560 (71) |
| HER2-low | 12260 (43) | 2435 (36) | 2680 (61) | 1837 (41) | 3526 (46) | 1157 (42) | 625 (29) |
| IHC 1+ | 8843 (31) | 1733 (26) | 1732 (40) | 1466 (32) | 2446 (32) | 949 (34) | 517 (24) |
| IHC 2+ ISH - | 3417 (12) | 702 (10) | 948 (22) | 371 (8) | 1080 (14) | 208 (7) | 108 (5) |
| **HER2 antibody used** |  | Dako (A0485) up to Jan 2005;  Thermo-scientific cerb-B2 SP3 (RM9103-S)  from Feb 2005 | 4B5 Ventana  (Roche) | 4B5 Ventana  (Roche) | 4B5 Ventana  (Roche) | Hercep Test (Dako) up to Feb 2012; HER2 4B5 (Roche) from Mar 2012 | 4B5 Ventana  (Roche) |
| **HER2 detection system used** |  | Ventana multimer detection kit | Ventana multimer detection kit | Ventana multimer detection kit | OptiView DAB IHC Detection Kit (Ventana) | Ventana multimer detection kit | I-VIEW (Ventana) |
| **Type of ISH used** |  | FISH | FISH | SISH | FISH | FISH from Jan 2009 to Feb 2012, DISH from March 2012 | FISH |

Abbreviation: JBCR, Singapore Health Services’ Joint Breast Cancer Registry; NTUH, National Taiwan University Hospital; SMC, Samsung Medical Centre; SNUH, Seoul National University Hospital; JFCR, Japanese Foundation for Cancer Research; NCCHE, National Cancer Centre Hospital East; IHC, immunohistochemistry; ISH-, in-situ hybridization non-amplified; FISH, Fluorescence in-situ hybridization; SISH, Silver in-situ hybridization; DISH, Dual in-situ hybridization.

**Table S2.** Sensitivity analysis of relapse-free survival and overall survival by HER2 and hormone receptor status

|  | **Sensitivity analysis^a^** | | | | | | |
| --- | --- | --- | --- | --- | --- | --- | --- |
|  | **Center-adjusted** | | |  | **Multivariable-adjusted^b^** | | |
|  | **Hazard ratio**  **(95% CI)** | ***P*** | ***P (int)*** |  | **Hazard ratio**  **(95% CI)** | ***P*** | ***P (int)*** |
| **Relapse-free survival** |  |  |  |  |  |  |  |
| Overall: HER2-low vs HER2-zero | 0.89 (0.83 - 0.95) | <0.001 | - |  | 0.93 (0.87 - 0.99) | 0.022 | - |
|  |  |  |  |  |  |  |  |
| Hormone receptor-positive: HER2-low vs HER2-zero | 0.94 (0.87 - 1.02) | 0.118 | 0.995 |  | 0.92 (0.86 – 0.99) | 0.038 | 0.829 |
| Hormone receptor-negative: HER2-low vs HER2-zero | 0.94 (0.82 - 1.08) | 0.378 |  |  | 0.94 (0.82 - 1.07) | 0.356 |  |
|  |  |  |  |  |  |  |  |
| **Overall survival** |  |  |  |  |  |  |  |
| Overall: HER2-low vs HER2-zero | 0.83 (0.76 - 0.91) | <0.001 | **-** |  | 0.88 (0.81 - 0.97) | 0.006 | - |
|  |  |  |  |  |  |  |  |
| Hormone receptor-positive: HER2-low vs HER2-zero | 0.93 (0.84 - 1.03) | 0.170 | 0.143 |  | 0.92 (0.83 - 1.02) | 0.096 | 0.151 |
| Hormone receptor-negative: HER2-low vs HER2-zero | 0.81 (0.68 – 0.95) | 0.012 |  |  | 0.80 (0.67 – 0.94) | 0.008 |  |

Abbreviation: int, interaction between HER2 and hormone receptor status.

a Patients diagnosed in 2000-2010 were censored at the maximum follow-up time of patients diagnosed in 2011-2015 (9.82 years)

b Covariates adjusted were study center, age at diagnosis, ethnicity, year of diagnosis, histology, overall stage, hormone receptor status, grade, received radiotherapy, endocrine therapy and chemotherapy.

**Figure S1.** Flowchart of patients included in the ABCCG cohort analysis.


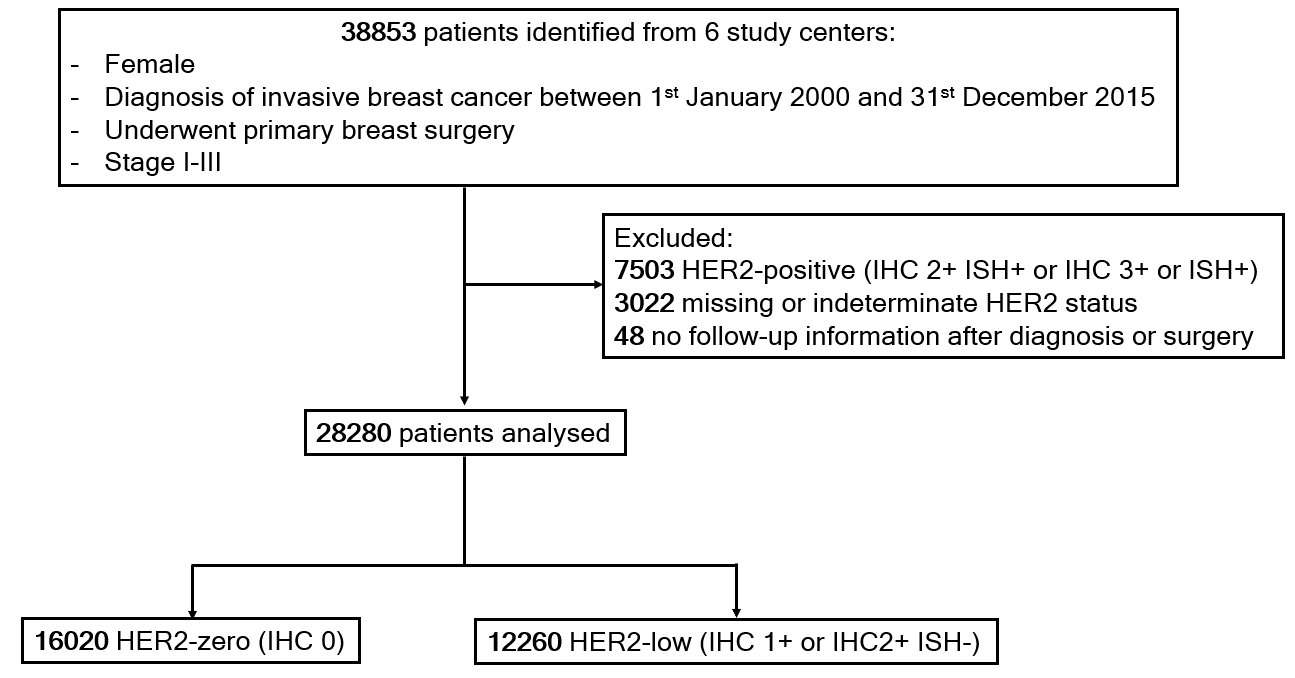


**Figure S2.** Estimated heterogeneity between study centers in (A) relapse-free survival, and (B) overall survival (based on pooling univariable HR estimate from each center with a random effect restricted maximum likelihood estimation model. HR, hazard ratio; I^2^, index of heterogeneity)

| 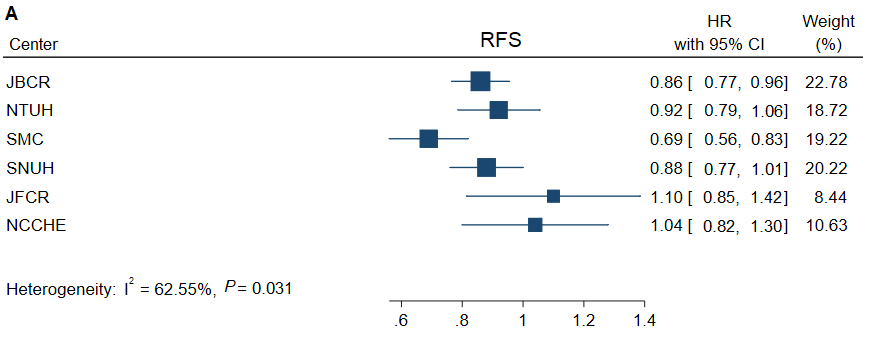  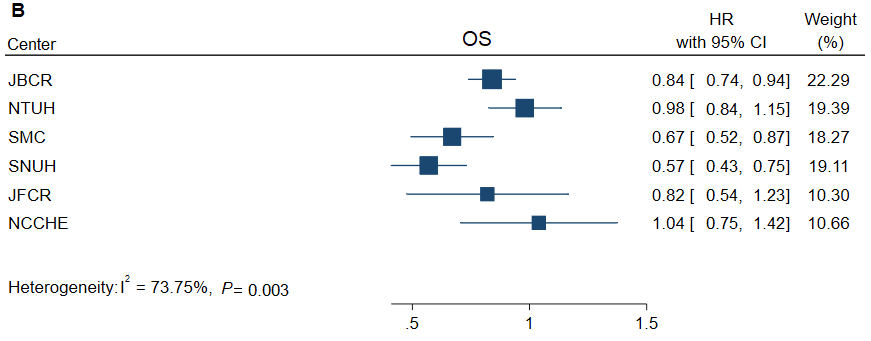 |
| --- |

**Figure S3.** Association of *ERBB2* mRNA expression with HER2 IHC and *ERBB2* CNV by hormone receptor status (TCGA-BRCA): (A-D) Hormone receptor-positive subgroup of TCGA-BRCA dataset: *ERBB2* mRNA expression by (A) HER2-low and HER2-zero, (B) HER2 IHC score, (C) *ERBB2* CNV; (D) *ERBB2* CNV against HER2 IHC. (E-H) Hormone receptor-negative subgroup of TCGA-BRCA dataset: *ERBB2* mRNA expression by (E) HER2-low and HER2-zero, (F) HER2 IHC score, (G) *ERBB2* CNV; (H) *ERBB2* CNV against HER2 IHC.


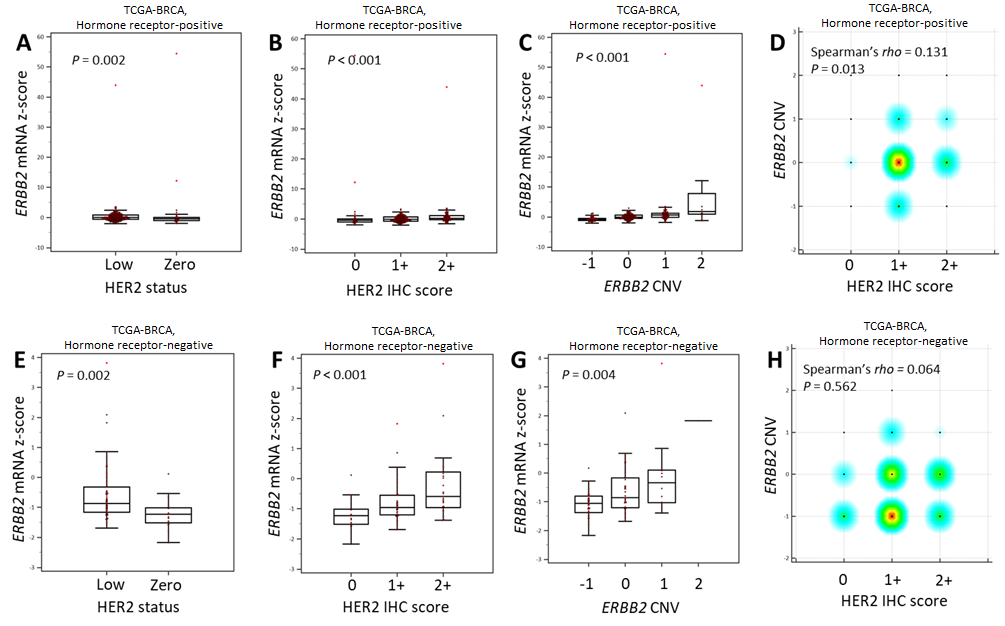


**Figure S4.** Relapse-free survival by *ERBB2* mRNA expression levels in combined TCGA-BRCA and METABRIC dataset (Quartile 1: lowest; Quartile 4: highest)


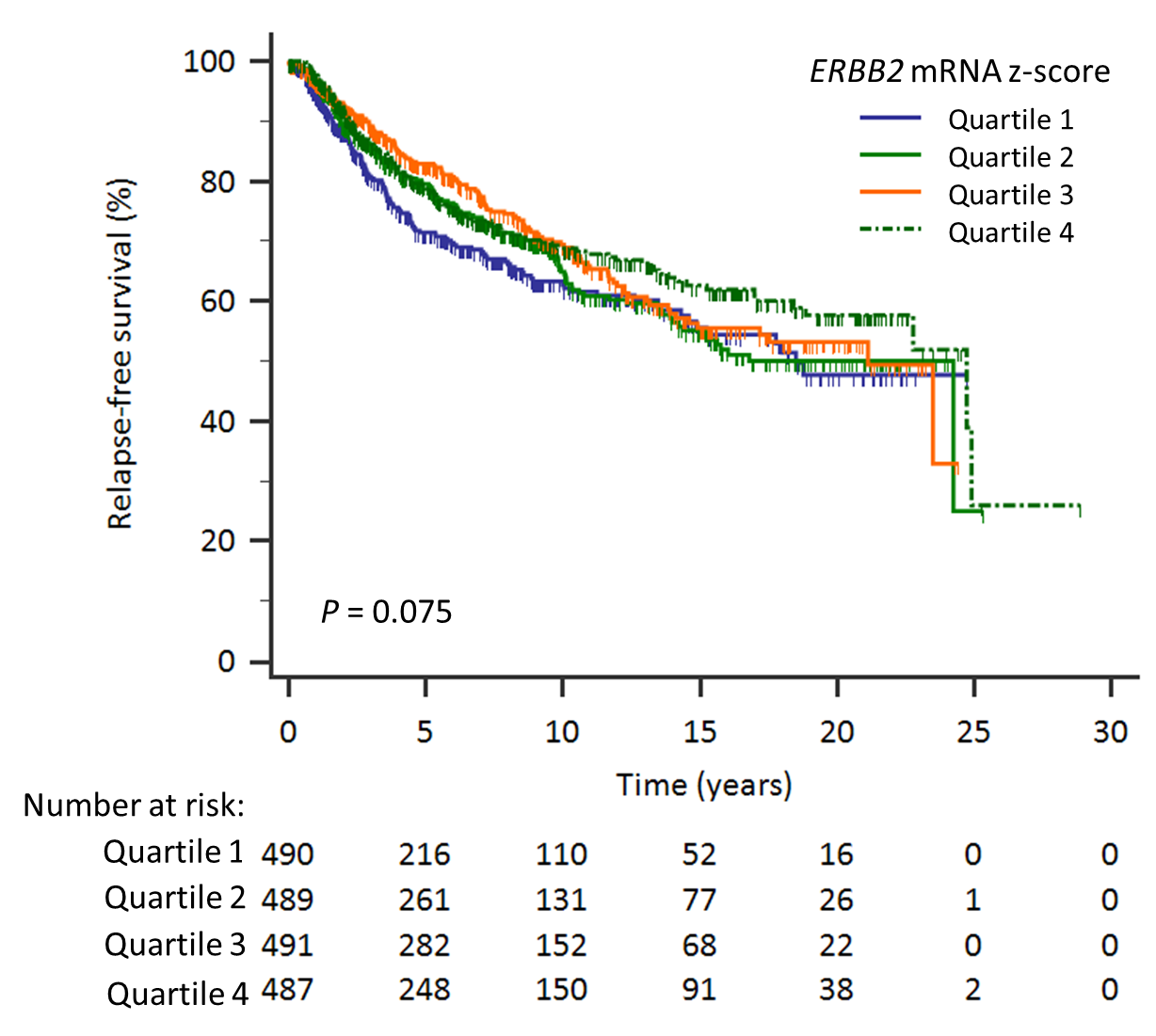

Supplement: Supplementary file 1 — Additional file 1: Table S1. Database information and details of HER2 testing by study centre. Table S2. Sensitivity analysis of relapse-free survival and overall survival by HER2 and hormone receptor status. Figure S1. Flowchart of patients included in the ABCCG cohort analysis. Figure S2. Estimated heterogeneity between study centres in (A) relapse-free survival, and (B) overall survival (based on pooling univariable HR estimate from each centre with a random effect restricted maximum likelihood estimation model. HR, hazard ratio; I2, index of heterogeneity). Figure S3. Association of ERBB2 mRNA expression with HER2 IHC and ERBB2 CNV by hormone receptor status (TCGA-BRCA): (A-D) Hormone receptor-positive subgroup of TCGA-BRCA dataset: ERBB2 mRNA expression by (A) HER2-low and HER2-zero, (B) HER2 IHC score, (C) ERBB2 CNV; (D) ERBB2 CNV against HER2 IHC. (E-H) Hormone receptor-negative subgroup of TCGA-BRCA dataset: ERBB2 mRNA expression by (E) HER2-low and HER2-zero, (F) HER2 IHC score, (G) ERBB2 CNV; (H) ERBB2 CNV against HER2 IHC. Figure S4. Relapse-free survival by ERBB2 mRNA expression levels in combined TCGA-BRCA and METABRIC dataset (Quartile 1: lowest; Quartile 4: highest) [file 12916_2022_2284_MOESM1_ESM.docx]
